# The left atrial appendage morphology and gender differences by multi-detector computed tomography in an Egyptian population

**DOI:** 10.1186/s43044-020-00072-2

**Published:** 2020-07-02

**Authors:** Mohammed Elzeneini, Ahmed Elshazly, Ahmed El Mahmoudy Nayel

**Affiliations:** Department of Cardiology, Ain Shams University, Cairo, Egypt

**Keywords:** Left atrial appendage, Left atrium, Cardiac computed tomography

## Abstract

**Background:**

The left atrial appendage (LAA) is the main source of thromboembolism in patients with non-valvular atrial fibrillation. Unique LAA morphologies have been associated with the risk of thromboembolism. This study investigates the LAA anatomy in the Egyptian population using cardiac multi-detector computed tomography (MDCT).

**Results:**

We included 252 consecutive patients presenting for coronary computed tomography angiography in 2 tertiary centers in Egypt in the period from January to July 2017. Patients with atrial fibrillation, valvular affection, or left ventricular dysfunction were excluded. Two and three-dimensional cardiac MDCT images were assessed for LAA morphology, volume, length, and orifice position. The distribution of LAA morphologies was windsock (32.5%), chicken wing (25.4%), cauliflower (22.6%), and cactus (19.4%). Differences in the LAA dimensions in the 4 morphological variants were described. Females were less likely to have a chicken wing LAA morphology compared to males (7.9% vs 34.7%, *p* value < 0.01), and had a larger LAA volume, smaller LAA length, and a higher prevalence of high LAA orifice position.

**Conclusions:**

The most common LAA morphology in our study population is windsock, which may represent the Egyptian population or patients in sinus rhythm. Females were less likely to have a chicken wing LAA morphology, and had a larger LAA volume, smaller length, and higher incidence of high orifice position. Clinical correlation into the translation of these differences into thromboembolic risk is required.

## Background

The left atrial appendage (LAA) is an anatomical finger-like projection extending from the left atrium (LA) with distinct anatomical and physiological properties independent from the LA [1, 2]. It lies anterior and lateral to the left pulmonary veins [3]. It is well recognized as a structure of high clinical significance, being the source of thromboembolism in more than 90% of patients with non-valvular atrial fibrillation presenting with stroke [4].

Different morphologies of the LAA have been described. Wang et al. put forward a classification of the LAA morphology into chicken wing, windsock, cactus, and cauliflower [5] and Kimura et al. later added quantitative measures to the classification to minimize subjectivity [6]. Clinical correlations found that non-chicken wing morphologies were associated with a higher risk of thromboembolic events than the chicken wing morphology [7, 8]. Compared with the chicken wing morphology, the cactus, windsock, and cauliflower morphologies were 4.08, 4.5, and 8.0 times more likely to have a stroke respectively [7], due to lower LAA flow velocities contributing to higher thrombus formation susceptibility [9]. Another study suggested a higher clinical significance of LAA orifice position in the risk of thromboembolism than LAA morphology [10]. These clinical correlations make an accurate understanding of the LAA anatomy important.

A lot of variability exists in the prevalence of different LAA morphologies in the literature. While a number of studies have shown the chicken wing morphology to be the most prevalent and cauliflower morphology the least prevalent [7, 11–13], other studies have shown the windsock [14] and cactus [15] morphology to be most prevalent. A number of factors could be contributing this variability including racial or demographic differences. In this study, we aimed to investigate the LAA morphology and dimensions in the Egyptian population, evaluated by cardiac multi-detector computed tomography (MDCT), and investigate gender differences in the LAA anatomy. These findings could help direct future clinical correlation studies, as well as help LAA occlusive device sizing [16].

## Methods

### Study population

The study population consisted of 252 consecutive patients presenting for outpatient coronary computed tomography angiography (CCTA) for exclusion of coronary artery disease at 2 tertiary centers in Egypt during the period from January to July 2017. The research protocol was approved by the ethics committee, and informed consent was obtained from all patients.

The following baseline data was collected: age, gender, history of valvular heart disease, heart failure, atrial fibrillation, and history of stroke or transient ischemic attack. Echocardiograms were reviewed for left ventricular function and presence of valvular heart disease, and electrocardiograms (ECGs) were reviewed for heart rate and rhythm. Exclusion criteria for performing CCTA included contrast allergy, renal impairment (creatinine > 2 mg/dl), active asthma, and weight greater than 140 kg. Patients with a history of atrial fibrillation or frequent premature beats on ECG that may result in image artifacts were excluded from the study, as well as patients with a history of heart failure, ejection fraction less than 50%, and moderate-to-severe valvular heart disease. CT images with motion artifacts that prevented adequate analysis of the left atrium were also excluded.

### Imaging

Cardiac MDCT imaging was performed using a 64-slice MDCT scanner (Aquilion 64, Toshiba Medical Systems, Japan) according to standard clinical protocol. Patients were examined in supine position, in a single breath hold, at a controlled heart rate of less than 70 beats per minute. Scanning parameters included tube current 400 mAs, potential 120 kV, rotation time 400 ms, and collimation 64 × 0.5 mm. All examinations were ECG-gated, and were conducted after administration of 50-80 ml of non-ionic iopromide contrast medium (Ultravist) at a rate of 4-6 ml/s. Axial source images were acquired in spiral mode, and two and three-dimensional reconstruction images were created at four segments at 40 to 70% of the R to R interval. The reconstructed images were processed on a separate work station (Vitrea) with multi-planar formatting and volume rendering to visualize individual heart structures with high detail. All images were analyzed by 2 expert cardiologists, who were blinded to patient history.

All images were evaluated for LA diameter and volume, as well as LAA volume, length, orifice position, and morphology. LA diameter was measured in its maximum anteroposterior dimension in axial images. LA and LAA volumes were measured from three-dimensional reconstruction images. The LAA orifice was identified as the narrowest part of the LAA opening, and its position was classified in relation of its superior aspect to the opening of the upper left pulmonary vein into high, medium, and low (above, in-line with, and below the opening of upper left pulmonary vein respectively). The LAA morphology was categorized based on Wang et al. and Kimura et al.’s previous classifications (5)(6) into 4 distinct morphological variants: windsock (having a dominant central lobe of length > 40 mm, and either secondary lobes arising in one direction or bending over 100°), chicken wing (having a dominant lobe > 40 mm with an acute bend of less than 100° in its proximal or middle part), cactus (having a dominant central lobe of length < 40 mm and secondary lobes arising in both superior and inferior directions), and cauliflower (having a length of < 40 mm with complex internal structure).

### Statistical analysis

The IBM SPSS software was used for statistical analysis. Continuous variables were presented as mean ± standard deviation while categorical variables were presented as numbers and percentages. Differences in the LA and LAA dimensions in the 4 morphologic types were compared using one-way analysis of variance (ANOVA) for continuous variables and chi-square test for categorical variables. Differences in the LA and LAA dimensions as well as LAA morphological types were then compared in males and females using Student’s *t* test for continuous variables and chi-square test for categorical variables.

## Results

**Fig. 1:**
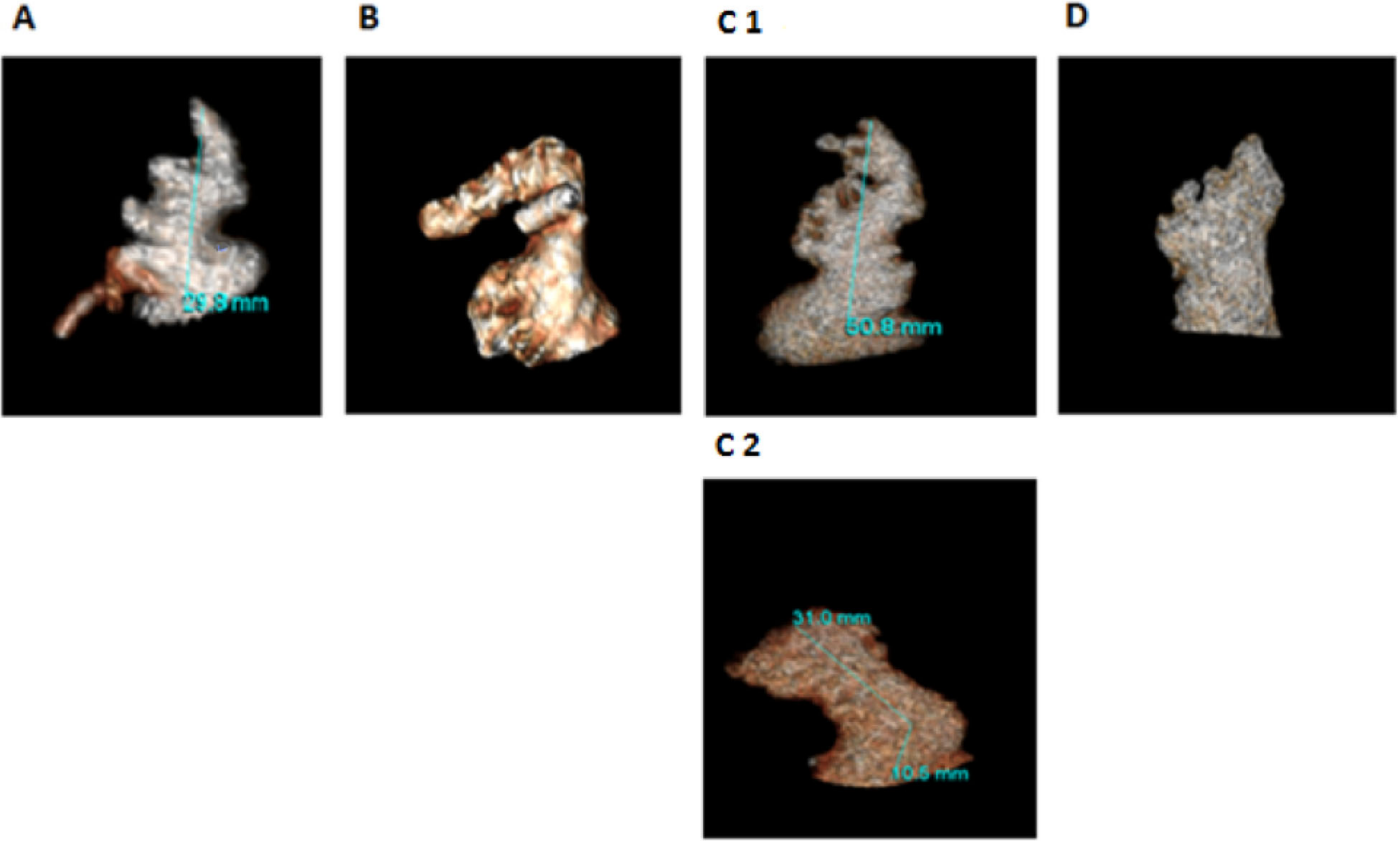
The four different left atrial appendage morphologies in our three-dimensional reconstruction MDCT images: cactus (**a**), chicken wing (**b**), windsock (**c1** and **c2**), and cauliflower (**d**)

**Fig. 2:**
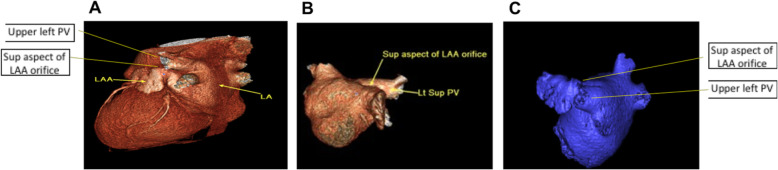
The three different left atrial appendage orifice positions in our three-dimensional reconstruction MDCT images: superior aspect of orifice is below (**a**), in-line with (**b**) or above (**c**) the opening of the upper left pulmonary vein

The mean age of our study population (*n* = 252) was 53.77 years ± 9.09. Almost two-thirds were males (65%, *n* = 164) and one-third were females (35%, *n* = 88). We identified the four distinct morphological variants of the LAA in our study population, using the criteria described in methods, demonstrated in our reconstruction images in Fig. 1. The distribution of these morphologies, in descending order of frequency, was as follows: windsock (32.5%, *n* = 82), chicken wing (25.4%, *n* = 64), cauliflower (22.6%, *n* = 57), and cactus (19.4%, *n* = 49). The mean LA diameter was 38.52 mm ± 4.86 and mean LA volume was 97.71 ml ± 31.01. The mean LAA volume was 7.83 ml ± 3.66 and mean LAA length was 39.31 mm ± 7.72. Half the patients had a low orifice below the left upper pulmonary vein (50.0%, *n* = 126), followed by a mid orifice in line with the left upper pulmonary vein (34.9%, *n* = 88) followed by a high orifice above the left upper pulmonary vein (15.1%, *n* = 38). Images of the 3 different LAA orifice positions in our three-dimensional reconstruction images are shown in Fig. 2. The distribution of the different LAA morphologies and mean LA and LAA dimensions are summarized in Table 1.

**Table 1:** Mean dimensions of the left atrium and left atrial appendage as measured by MDCT, and the distribution of the four different left atrial appendage morphologies

		*N* = 252	
Left atrial diameter, mm		Mean ± SD	38.52 ± 4.86
		Range	26-48
Left atrial volume, ml		Mean ± SD	97.71 ± 31.01
		Range	39_182
LAA volume, ml		Mean ± SD	7.83 ± 3.66
		Range	3-16
LAA length, mm		Mean ± SD	39.31 ± 7.72
		Range	24-53
LAA orifice position (*N* = 252)	Low	*N* = 126 (50.0%)	
	Mid	*N* = 88 (34.9%)	
	High	*N* = 38 (15.1%)	
LAA morphology (*N* = 252)	Windsock	*N* = 82 (32.5%)	
	Chicken wing	*N* = 64 (25.4%)	
	Cactus	*N* = 49 (19.4%)	
	Cauliflower	*N* = 57 (22.6%)	

*LAA* left atrial appendage, *SD* standard deviation

Table 2 summarizes the differences in the LA and LAA dimensions in the 4 morphologic types of the LAA. The cactus morphology was associated with the largest LA diameter but smallest LA volume. The windsock morphology was associated with the largest LA and LAA volumes. The cauliflower morphology was associated with the smallest LA diameter while the chicken wing morphology was associated with the smallest LAA volume. The majority of the cactus (87.8%) and a higher percentage of the windsock (53.7%) LAA morphologies were associated with a low appendage orifice, while a mid appendage orifice was the more common position in the chicken wing (50.0%) and cauliflower (43.9%) LAA morphologies.

**Table 2:** Differences in the left atrium and left atrial dimensions in the 4 morphological types of the left atrial appendage

		Windsock	Chicken wing	Cauliflower	Cactus	*P* value
		*N* = 82	*N* = 64	*N* = 57	*N* = 49	
Left atrial diameter, mm (mean ± SD)		39.10 ± 5.25	38.16 ± 4.65	37.14 ± 4.45	39.63 ± 4.60	0.03
Left atrial volume, ml (mean ± SD)		104.3 ± 29.57	99.06 ± 37.23	95.28 ± 30.85	87.65 ± 20.88	0.02
LAA orifice position (*N*, %)	Low	44 (53.7%)	26 (40.6%)	13 (22.8%)	43 (87.8%)	< 0.01
	Mid	25 (30.5%)	32 (50.0%)	25 (43.9%)	6 (12.2%)	
	High	13 (15.9%)	6 (9.4%)	19 (33.3%)	0 (0.0%)	
LAA volume, ml (mean ± SD)		10.06 ± 3.61	6.27 ± 3.23	7.44 ± 3.30	6.61 ± 2.89	< 0.01
LAA length, mm (mean ± SD)		45.40 ± 3.86	43.95 ± 5.35	31.53 ± 3.20	32.12 ± 4.46	< 0.01

Categorical variables were compared by chi-square test, continuous variables were compared by one way ANOVA

*LAA* left atrial appendage, *SD* standard deviation

Differences in the LAA anatomy with respect to age and gender were investigated. There was no difference in mean patient age in the 4 different LAA morphological variants. There were however significant differences between males and females. Females were much less likely to have a chicken wing LAA morphology compared to males whose pre-dominant morphology was chicken wing (7.9% vs 34.7%, *p* value < 0.01). Table 3 shows the differences in the LA and LAA dimensions as well as LAA morphological types between males and females. There was no significant difference in LA dimensions between males and females but there were significant differences in LAA dimensions. Females had a larger mean LAA volume compared to males (8.56 ml vs 7.47 ml, *p* value 0.026), a smaller mean LAA length (37.91 mm vs 40.02 mm, *p* value 0.038), and a higher prevalence of high LAA orifice (29.6% vs 7.4%, *p* value < 0.01).

**Table 3:** Differences in the left atrium and left atrial appendage dimensions as well as left atrial appendage morphological types between males and females

		Sex		
		Male (*N* = 164)	Female (*N* = 88)	*P* value
Left atrial diameter, mm (mean ± SD)		38.73 ± 5.24	38.1 ± 4.11	0.333
Left atrial volume, ml (mean ± SD)		97.76 ± 28.73	97.82 ± 35.21	0.988
LAA volume, ml (mean ± SD)		7.47 ± 3.44	8.56 ± 4.09	0.026
LAA length, mm (mean ± SD)		40.02 ± 6.86	37.91 ± 8.93	0.038
LAA orifice position (*N*, %)	Low	76 (46.3%)	50 (56.8%)	< 0.01
	Mid	76 (46.3%)	12 (13.6%)	
	High	12 (7.4%)	26 (29.6%)	
LAA morphology (*N*, %)	Windsock	52 (31.7%)	30 (34.1%)	< 0.01
	Chicken wing	57 (34.7%)	7 (7.9%)	
	Cauliflower	36 (21.9%)	21 (23.8%)	
	Cactus	19 (11.5%)	30 (34.1%)	

Categorical variables were compared by chi-square test, continuous variables were compared by Student’s *t* test

*LAA* left atrial appendage, *SD* standard deviation

## Discussion

### LAA morphology

The distribution of the four different LAA morphologies in our study population was windsock, followed by chicken wing, followed by cauliflower, followed by cactus. This distribution of LAA morphologies is similar to Korhonen et al.’s study on patients in Finland with ischemic stroke of cryptogenic or cardiac etiology other than atrial fibrillation [14]. On the other hand, it disagrees with a lot of studies conducted on the LAA that showed the chicken wing morphology to be the most common and cauliflower to be the least common, most of which were conducted on patients with atrial fibrillation undergoing catheter ablation. These studies include those conducted by Di Biase et al. [7], Bai et al. [11], Anselmino et al. [12], and Hirata et al. [13]. Other studies found different distributions, including Fukushima et al. on patients in Japan where cactus morphology was found to be most prevalent [15]. This variability in the distribution of LAA morphologies may be due racial or demographic differences in different populations. An important observation is that the study population in most studies that showed a high prevalence of the chicken wing LAA morphology were in atrial fibrillation [7, 11–13], while our study population as well as Korhonen et al.’s study population were in sinus rhythm [14]. This suggests a possible association between the presence of atrial fibrillation and the chicken wing LAA morphology.

### LAA dimensions

The mean LAA volume measured in our study population (7.8 ml) is smaller than that measured in Wang et al.’s study (8.8 ml) [5], Kimura et al.’s study (16.1 ml) [6], and Korhonen et al.’s study (12.6 ml) [14]. The mean LAA length (39.3 mm) is also smaller than in Wang et al.’s study (45.8 mm) [5]. This may suggest that a smaller mean LAA volume and length is present in the Egyptian population compared to other populations, which is of significance due to the increasing evidence that a larger LAA volume promotes blood stasis and contributes to a higher risk of stroke [17, 18]. A low LAA orifice position in relation to the left upper pulmonary vein is also more prevalent in our study population (50.0%) compared to Wang et al.’s study which showed a higher prevalence of mid (58.1%) and high (30.2%) orifice positions [5]. This is significant due to the literature evidence that a higher LAA orifice position is associated with a higher risk of stroke, due to slower blood flow contributing to thrombosis [10, 19]. While these anatomical characteristics might suggest a “more favorable” LAA anatomy present in the Egyptian population compared to others, further studies with correlation with thromboembolic risk are needed.

### Age and gender considerations

Our results showed a significant association between gender and the LAA anatomy. Females were less likely to have a chicken wing morphology compared to males. Females also had a larger LAA volume, a smaller LAA length, and a higher prevalence of high LAA orifice position. While these features have been associated in some studies to a higher thromboembolic risk [7, 10, 17], further studies of clinical correlation into the translation of these differences into thromboembolic risk is required. There is paucity of data on gender differences in LAA morphologies. Korhonen et al. had reported an association between female gender and a shorter LAA length that was lost after adjusting for body surface area, and was attributed to the different amounts of pericardial fat contributing to atrial remodeling [20]. No significant association was found between age and the LAA morphology in our study. Hirata et al. also found no significant association in patients in sinus rhythm but found a significant increase in the chicken wing morphology in older age groups in patients with atrial fibrillation. This could be explained by aging in atrial fibrillation patients affecting remodeling of the left atrial appendage wall [13].

## Conclusions

The most common LAA morphology in our study population is windsock, which may represent the Egyptian population or the predominant morphology in patients in sinus rhythm. Females are more likely to have a non-chicken wing LAA morphology, a larger LAA volume, a smaller length, and a higher incidence of high orifice position than males. Clinical correlation into the translation of these differences into thromboembolic risk is required.

## Data Availability

All data and material used can be provided upon request.

## Abbreviations

CCTA: Cardiac computed tomography angiography
ECG: Electrocardiogram
LA: Left atrium
LAA: Left atrium appendage
MDCT: Multi-detector computed tomography
